# Hematological abnormalities before and after initiation of cancer treatment among breast cancer patients attending at the University of Gondar comprehensive specialized hospital cancer treatment center

**DOI:** 10.1371/journal.pone.0271895

**Published:** 2022-08-08

**Authors:** Melak Aynalem, Nurayni Adem, Firdews Wendesson, Bewket Misganaw, Simegnew Mintesnot, Nega Godo, Solomon Getawa, Tiruneh Adane, Berhanu Woldu, Elias Shiferaw

**Affiliations:** 1 Department of Hematology and Immunohematology, School of Biomedical and Laboratory Sciences, College of Medicine and Health Sciences, University of Gondar, Gondar, Ethiopia; 2 Department of Medical Laboratory Science, School of Biomedical and Laboratory Sciences, College of Medicine and Health Sciences, University of Gondar, Gondar, Ethiopia; Oregon State University, UNITED STATES

## Abstract

**Background:**

Breast cancer is the most frequent and fatal cancer type globally. The fatality rate of breast cancer is mostly due to disease complications like hematological alterations. Therefore, this study aimed to assess the hematological abnormalities before, during, and after the initiation of cancer treatment in breast cancer patients at the University of Gondar comprehensive specialized hospital.

**Methodology:**

Hematological profiles were collected from 267 breast cancer patients who attended the cancer treatment center from September 2017 to August 2021. A data extraction sheet was used to extract data from the patient’s medical chart, including sociodemography, clinical, and hematological profiles. EPI info version 3.5.1 and SPSS Version 25 softwares were used for data entrance and analysis, respectively. Descriptive statistics were summarized using frequency and percentage. The Friedman test followed by a Wilcoxon signed rank test was used to compare the mean difference between the hematological profiles at zero and after the 4^th^ and 8^th^ cycles of treatment.

**Result:**

Of the total participants, 91% were females, and the median age of the study participants was 45 (IQR = 36, 55) years. Red blood cell, white blood cell, and lymphocyte counts, as well as hematocrit and hemoglobin values, were significantly reduced after the initiation of cancer treatment, while the platelet count and red cell distribution width were significantly increased. The prevalence of anemia was 21.7% (95% CI: 16.6, 26.8), 22.7% (95% CI: 17.6, 27.8), and 26.4% (95% CI: 21.3, 31.5) before, during, and after the initiation of cancer treatment, respectively. The prevalence of leukopenia before, during, and after treatment was 9.7%, 18.8%, and 15.1%, respectively. Finally the prevalence of thrombocytopenia was 6.3%, 3.4%, and 8% at before, during, and after treatment, respectively.

**Conclusion:**

This study concluded that many hematological parameters were significantly affected by the breast cancer treatment. Therefore, proper patient follow-up and provide appropriate interventions related to their hematological abnormalities is crucial. It is also important to conduct further prospective studies to confirm the findings of this study.

## Background

Breast cancer is the most common cancer type globally [1, 2]. It affects both developing and developed countries. Breast cancer frequently occurs at a young age and it is the leading cause of mortality in a woman’s life [3]. Globally, about 2.1 million cases were diagnosed in 2018, with more than 560,000 deaths. In Africa and East Africa, the total incidence of breast cancer was 168,690 and 40,310, respectively [4]. In Ethiopia, breast cancer incidence is rising and has become the most common cancer in women, causing high rates of morbidity and mortality [5].

The causes of the morbidity and mortality rate in breast cancer are respiratory failure, cardiopulmonary arrest, and multi-system organ failure [6]. Besides, hematological abnormalities are common complications of breast cancer patients [7]. Hematological abnormalities can be observed both before and after cancer treatment. Pretreatment hematological abnormalities are mostly due to suppression of hematopoiesis via bone marrow infiltration [8, 9]. Several studies confirm that pretreatment anemia, neutropenia, and thrombocytopenia were found to be common in breast cancer patients [10, 11]. The post-treatment hematological abnormalities are associated not only with the malignant cell effect, but also with the chemotherapy and radiotherapy medications administered to the patients [12, 13]. From hematological abnormalities, anemia is the most commonly found in breast cancer patients [14]. Tumor-related bleeding, tumor invasion of bone marrow, malnutrition caused by the tumor, abnormal iron metabolism, renal physiology impairment, and compromised bone marrow function are the possible mechanisms for the development of anemia [15, 16]. Besides, leucopenia is mostly associated with medications that cause myelosuppression [17]. Low platelet counts in breast cancer are mostly associated with the cancer cells that cause frequent activation of the coagulation system, which can manifest as thrombocytopenia [18]. In addition, thrombocytosis was also associated with poor progression. -free survival [19, 20].

Breast cancer is a public health problem in Ethiopia. However, there is limited research conducted on breast cancer and its effects on the hematological profile. Therefore, the current study aimed to assess hematological abnormalities in breast cancer patients before, during, and after the initiation of cancer treatments at the University of Gondar Comprehensive Specialized Hospital (UoG-CSH).

## Methods and materials

A retrospective cohort study was conducted at the UoG-CSH cancer treatment center from September 2017 to August 2021. The hospital is located in Gondar town, which is 750 kilometers northwest of the capital city of Ethiopia, Addis Ababa. The town has a latitude and longitude of 12° 36´ N and 37° 28´ E with an elevation of 2133 meters above sea level. The estimated population size in 2020 was 362,000. The town currently has one hospital, UoG-CSH, which serves more than seven million residents from the town and neighboring zones and regions of Ethiopia.

All breast cancer patients who had visited the UoG-CSH cancer treatment center were considered as a source population, and breast cancer patients who had visited the UoG-CSH cancer treatment center during the study period and had a complete data were considered as a study population. The hematological parameters of the patients were considered as dependent variables, and the socio-demographic variables (age, gender, and residence) and clinical variables (type of cancer, treatment regime, comorbidity, and stage of breast cancer) were taken as independent variables. Study participants with incomplete sociodemographic, clinical, and laboratory data were excluded from the study.

Data on socio-demographics, clinical characteristics, and laboratory test results were extracted from the patients medical records by using a data extraction sheet. Furthermore, data on the patient’s hematological parameters were collected before treatment initiation (zero cycles), during treatment (at the end of the 4^th^ cycle), and after completion of treatment (at the end of the 8^th^ cycle). To assure the quality of data, training was given to data collectors, and they were closely supervised by the investigators during data collection. Then, the completeness and consistency of data were checked daily. Finally, a double entry was performed to assure the validity of the data.

### Statistical analysis

The data was entered into Epi-info version 3.5.1 and transferred to SPSS version 25 (IBM Corporation, Armonk, NY, USA) software for analysis. Descriptive statistics were summarized using frequency and percentage. The data was tested for normality with the Shapiro-Wilk and Kolmogorov-Smirnov tests. The mean and standard deviation (SD) were used for normally distributed data and the median and interquartile range (IQR) for skewed data. The Friedman test was used to compare the mean difference between the hematological profiles before, during, and after the initiation of cancer treatment. Further, Wilcoxon analysis was used for pairwise comparisons. A P-value of ≤ 0.05 was considered statistically significant.

### Ethical considerations

In this retrospective study, obtaining informed consent was not possible. So, we were granted a waiver of consent by the Ethical Review Committee, and patient medical records were anonymized and de-identified before analysis. The study protocol was reviewed by the ethical review committee of the School of Biomedical and Laboratory Sciences, College of Medicine and Health Sciences, University of Gondar (Ref. No./SBLS/2897/2013). A permission letter was obtained from the UoG-CSH medical director and cancer treatment center. A code was used to ensure the confidentiality of data. Any unauthorized person had no access to the collected data.

## Results

### Sociodemographic and clinical characteristics

As shown in Table 1, a total of 267 study participants with different stages of breast cancer were included in this study. The median age of breast cancer patients was 45 (IQR = 36, 55) years, and 55.4% were in the age group of 18 to 45 years. Of the participants, 91% and 63.3% were females and urban residents, respectively. Regarding the stage of cancer, 0.54%, 13.93%, 46.77%, and 38.8% were stage I, II, III, and IV, respectively. Considering the anatomical site of the breast cancer, about 47.7%, 48.95%, and 3.35% were developed on the left, right, and both breasts, respectively. The frequencies of liver, lung, bone, and lymph node metastasis were 15.7%, 21%, 4.9%, and 3.7%, respectively. Four patients (1.1%) had intestinal parasites, 1.1% had diabetes mellitus, and six (2.2%) had toxic goiter.

**Table 1:** Socio demographic and clinical characteristics of breast cancer patients at UoG-CSH, Northwest Ethiopia, 2021.

Variables	Categories	Frequency	Percentage
**Gender**	Male	24	9%
	Female	243	91%
**Age**	18–45	148	55.4%
	46–65	85	31.8%
	>65	34	12.7%
**Residence**	Urban	169	63.3%
	Rural	98	36.7%
**Stage of the cancer (n = 201)**	Stage I	1	0.54%
	Stage II	28	13.93%
	Stage III	94	46.77%
	Stage IV	78	38.8%
**Anatomical site of the cancer (n = 239)**	Left Breast	114	47.7%
	Right Breast	117	48.95%
	Both	8	3.35%
	Missed	28	10.5%
**Metastasis**	Metastasis to Liver	42	15.7%
	Metastasis to Lung	56	21%
	Metastasis to Bone	13	4.9%
	Metastasis to Lymph node	10	3.7%
**Presence of Co-morbidities**	Co-infection with IP	4	1.5%
	Co-infection with DM	3	1.1%
	Co-infection with Toxic Goiter	6	2.2%
	Other co-infection	53	19.9%

**Note:** Other co-infection: includes TB, HCV, and HBV.

### Comparison of hematological parameters before, during, and after cancer treatment

Hemoglobin concentration before, during, and after treatment was 14.30 ± 9.92, 13.96 ± 8.90, and 12.74 ± 2.45 g/dl, respectively. The white blood cell (WBC) count before, during, and after treatment was 6.56 ± 2.39, 5.53 ± 2.22, and 6.23 ± 3.48, respectively. Before, during, and after treatment, the platelet count was 287.56 102.27, 376.40 135.34, and 306.91 127.25, respectively (Table 2).

**Table 2:** Comparison of hematological parameters before and after treatment among breast cancer patients at UoG-CSH, Northwest Ethiopia, 2020.

Hematological parameters	Before treatment	During treatment	After treatment	p-value
	Mean ± SD	Mean ± SD	Mean ± SD	
**RBC**	5.76 ± 9.74	4.57 ± 0.54	4.66 ± 0.81	0.036*
**Hgb**	14.30 ± 9.92	13.96 ± 8.90	12.74 ± 2.45	0.020*
**HCT**	39.8 ± 7.13	38.29 ± 6.65	39.05 ± 6.66	0.000*
**MCV**	85.48 ± 10.46	84.84 ± 7.50	85.66 ± 8.53	0.009*
**MCH**	30.03 ± 17.42	28.29 ± 3.90	29.23 ± 6.12	0.181
**MCHC**	34.06 ± 17.59	32.64 ± 5.31	32.48 ± 4.30	0.119
**WBC**	6.56 ± 2.39	5.53 ± 2.22	6.23 ± 3.48	0.000*
**Neutrophile**	3.42 ± 1.60	3.37 ± 2.24	3.60 ± 3.34	0.236
**Lymphocyte**	2.13 ± 0.70	1.71 ± 0.89	2.62 ± 5.14	0.000*
**Mixed**	0.35 ± 0.07	0.75 ± 0.21	0.55 ± 0.49	0.368
**Platelet**	287.56 ± 102.27	376.40 ± 135.34	306.91 ± 127.25	0.000*
**RDW**	44.94 ± 4.38	45.81 ± 11.03	45.77 ± 6.96	0.008*
**MPV**	8.71 ± 1.25	8.69 ± 1.27	7.92 ± 1.80	0.470

Note

*P value less than 0.05 is a significance level.

**Abbreviations**: Hgb: Hemoglobin, WBC: White blood cell, RBC: Red blood cell, HCT: Hematocrit, MCV: Mean corpuscular volume, MCH: Mean corpuscular hemoglobin, MCHC: Mean corpuscular hemoglobin concentration, RDW: Red cell distribution width, MPV: Mean platelet volume, IQR: Interquartile range, SD: Standard deviation.

The red blood cell (RBC), Hemoglobin, hematoctite (HCT), WBC, lymphocyt, and mean cell volume (MCV) were significantly lower after treatment. Platelet count and RDW were significantly higher after treatment than before (P-value 0.001) (Table 3).

**Table 3:** Wilcoxon rank test for the comparison of hematological parameters before, during, and after treatment among breast cancer patients at UoG-CSH, Northwest Ethiopia, 2020.

Hematological parameters	p-value		
	Before to during treatment	Before to after treatment	During to after treatment
**RBC**	0.134	0.650	0.177
**Hgb**	0.001*	0.095	0.421
**HCT**	0.001*	0.038	0.408
**MCV**	0.001*	0.228	0.917
**RDW**	0.000*	0.164	0.153
**WBC**	0.000*	0.003*	0.318
**Lymphocyte**	0.000*	0.000*	0.000*
**Platelet**	0.000*	0.394	0.000*

Note

* significant level, P value less than 0.017 is a significance level.

**Abbreviations**: Hgb: Hemoglobin, WBC: White blood cell, RBC: Red blood cell, HCT: Hematocrit, MCV: Mean corpuscular volume, MCH: Mean corpuscular hemoglobin, MCHC: Mean corpuscular hemoglobin concentration, RDW: Red cell distribution width.

### Hematological abnormalities among breast cancer patients

**Fig 1:**
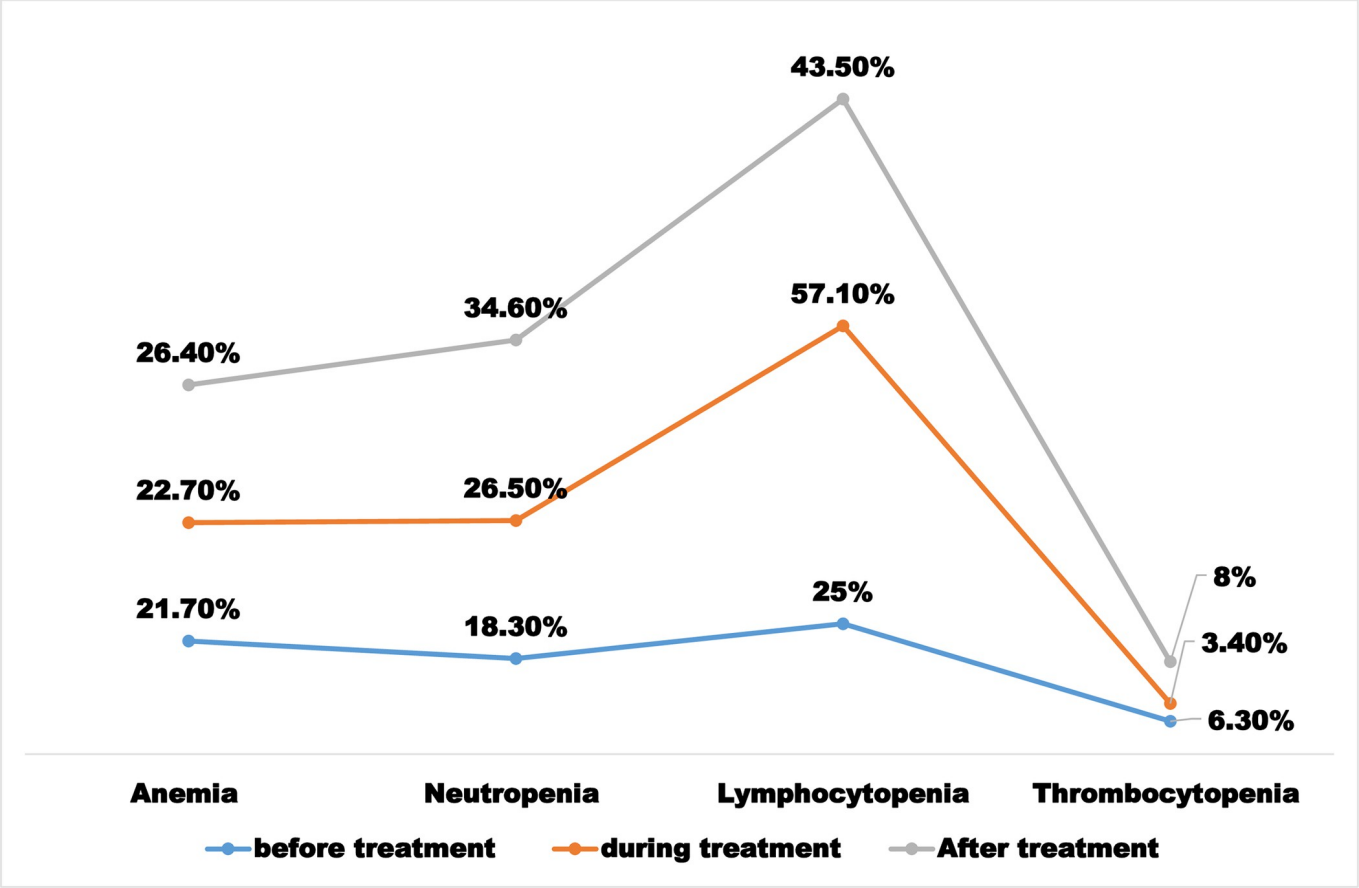
Hematological abnormalities with in the treatment cycle in breast cancer patients at UoG-CSH, Northwest Ethiopia, 2021.

The prevalence of anemia in breast cancer patients before, during, and after treatment was 21.7% (95% CI: 16.6, 26.8), 22.7% (95% CI: 17.6, 27.8), and 26.4% (95% CI: 21.3, 31.5), respectively. The frequency of leukopenia before, during, and after treatment was 9.7%, 18.8%, and 15.1%, respectively. The prevalence of leukocytosis was 7.7%, 6.3%, and 7%, respectively. We found that 6.3%, 3.4%, and 8% of breast cancer patients were thrombocytopenic before, during, and after treatment, respectively. Thrombocytosis before, during, and after treatment was 11.5%, 23.3%, and 10.3%, respectively (Table 4 and Fig 1).

**Table 4:** Hematological parameters of breast cancer patients at UoG-SCH cancer treatment center, Northwest Ethiopia, 2021.

Hematological	Categories	Before treatment	At 4^th^ cycle	At 8^th^ cycle
Parameters		N (%)	N (%)	N (%)
**Hgb**	Low	56 (21.7)	40 (22.7)	23 (26.4)
	Normal	196 (76.0)	134 (76.1)	63 (72.4)
	High	6 (2.3)	2 (1.1)	1 (1.1)
**MCV**	Low	36 (14.4)	24 (14.0)	13 (14.9)
	Normal	209 (83.6)	147 (86.0)	72 (82.8)
	High	5 (2.0)	0 (0)	2 (2.3)
**MCH**	Low	56 (22.5)	32 (18.7)	20 (23.0)
	Normal	173 (69.5)	124 (72.5)	58 (66.7)
	High	20 (8.0)	15 (8.8)	9 (10.3)
**MCHC**	Low	55 (22.1)	34 (19.9)	19 (22.1)
	Normal	184 (73.9)	129 (75.4)	63 (73.3)0
	High	10 (4.0)	8 (4.7)	4 (4.7)
**WBC count**	Low	25 (9.7)	33 (18.8)	13 (15.1)
	Normal	214 (82.6)	132 (75.0)	67 (77.9)
	High	20 (7.7)	11 (6.3)	6 (7.0)
**Neutrophil**	Low	30 (18.3)	30 (26.5)	18 (34.6)
	Normal	120 (73.2)	78 (69.0)	28 (53.8)
	High	14 (8.5)	5 (4.4)	6 (11.5)
**Lymphocyte**	Low	53 (25.0)	72 (57.1)	27 (43.5)
	Normal	156 (73.6)	53 (42.1)	32 (51.6)
	High	3 (1.4)	1 (0.8)	3 (4.8)
**Platelet**	Low	16 (6.3)	6 (3.4)	7 (8.0)
	Normal	208 (82.2)	129 (73.3)	71 (81.6)
	High	29 (11.5)	41 (23.3)	9 (10.3)

**Abbreviations**: Hgb: Hemoglobin, WBC: White blood cell, MCV: Mean corpuscular volume, MCH: Mean corpuscular hemoglobin, MCHC: Mean corpuscular hemoglobin concentration.

## Discussion

Breast cancer is a major public health problem worldwide with a rapidly increasing incidence. In order to enhance treatment effectiveness and patient survival, hematological profile data is a crucial characteristic that should be considered in patient care [21]. Therefore, the current study focused on assessing the hematological profiles in breast cancer patients.

This study found that most of the red blood cell parameters, such as RBC count, HCT, hemoglobin, and MCV, were significantly reduced from the initiation to the completion of breast cancer treatment, while the red cell distribution width (RDW) was significantly increased from pretreatment to post-treatment. This indicates that the degree of anemia was increased throughout treatment. Furthermore, the reduced MCV and increased RDW indicate that there is a high proportion of microcytic cells with a wide cell size distribution. This could be a side effect of cancer therapy, which may cause cell hemolysis, resulting in low RBC count and MCV with high RDW [8]. Besides, the significant reduction of RBC parameters was seen in the zero to fourth treatment cycle but not in the post-treatment period, which reveals that these groups are more susceptible to the treatment side effects. This can be due to patients with post-treatment being more commonly transfused with blood and blood products, so a significant cell reduction is not expected from pre-to-post treatment. In addition, the prevalence of anemia before, during, and after treatment was 21.7% (95% CI: 16.6, 26.8), 22.7% (95% CI: 17.6, 27.8), and 26.4% (95% CI: 21.3, 31.5), respectively. The prevalence of anemia in the current study was significantly increased from pretreatment to post-treatment. This finding is similar to a study conducted in Iran that showed anemia was lower before treatment (41%), than after treatment (43.1%), but the prevalence of anemia was higher than the current study result [22]. Also, our study is comparable with a study conducted in China, with the prevalence of pretreatment anemia being 25.3%, which increased during treatment [23]. In India, 60% of breast cancer patients showed pretreatment anemia, which was higher than the result of the current study [24]. This inconsistency in the findings could be due to sample size variation, differences in study set up, genetic factors, and lifestyle, which may explain the variation in anemia found in those studies.

In this study, most of the WBC parameters, such as total WBC count and lymphocyte counts, were significantly decreased from pre-treatment to post-treatment. Besides, the significant reduction in total WBC count was seen in the 0–4^th^ cycle and the 0–8^th^ cycle, while the lymphocyte count was significantly reduced in all the treatment cycles. The reduction of the WBC parameter can be related to breast cancer treatments. Simultaneously, the drugs cause harm to normal tissues, including parts of the immune system and immune cells like lymphoctes, which results in decreased immunological function and a lower chance of survival [25–27]. This finding is similar to the study conducted in Korea, which showed that WBC parameters were reduced through treatment [24]. However, this finding is divergent from the results of a study conducted in Nigeria, which found that 31.9% was before treatment and decreased to 11% during treatment [27]. This may be due to the difference in study population and design.

The current study found that platelet count was significantly increased before and after treatment, with a significant increase between the zero cycle and the fourth cycle, and between the fourth and the eighth cycle. This can be due to cancer cells’ ability to cause thrombocytosis and platelet aggregation [28–30]. Moreover, thrombocytopenia in the current study before treatment was 6.3% (95% CI: 3.3, 10.5) and after treatment was 8% (95% CI: 3.7, 12.3). This finding showed thrombocytopenia was increased from pretreatment to post-treatment. Studies have suggested that thrombocytopenia occurs in cases of breast cancer progression, particularly in bone metastasis. The current study finding was supported by a finding from a Philippine study which showed that thrombocytopenia was minimal among non-metastatic breast cancer patients [31]. Thrombocytosis was lower before (11.5%) and after (10.4%) treatment but significantly higher during treatment (23.3%). This finding is similar to the study conducted in Ethiopia, which showed that the prevalence of thrombocytopenia was 23.5% [21]. In contrast to this study, a study conducted in Switzerland revealed that thrombocytosis was increased throughout the treatment [11]. This variation could be due to the sample size, clinical characteristics, and socio-demographic differences between the study groups.

This study has its own limitations. The first limitation of this study is the retrospective nature of the study design. As a result, it is difficult to give a solid conclusion on the outcomes at a different stage of treatment.

## Conclusion

Breast cancer patients showed reduced levels of most RBC and WBC parameters throughout the treatment cycle. During the treatment cycle, platelet count and RDW increased. Therefore, breast cancer patients’ health follow-up should include a hematological profile test in each treatment cycle. Also, healthcare providers should be aware of hematological complications in cancer treatment. It is also important to conduct further prospective studies that can confirm the findings of this study.

## Supporting information

S1 Table Socio demographic clinical data collection sheet. (DOCX)

## List of abbreviations

HCT: hematoctite
IQR: Interquartile range
MCV: Mean Cell Volume
RBC: Red Blood Cell
RDW: red cell distribution width
SD: Standard Deviation
WBC: White Blood Cell
UoG-CSH: University of Gondar Comprhensive and Specialized Hospital
