# Supplementary material for: Hematological abnormalities before and after initiation of cancer treatment among breast cancer patients attending at the University of Gondar comprehensive specialized hospital cancer treatment center
Source: PLoS One. 2022 Aug 8;17(8):e0271895. doi: 10.1371/journal.pone.0271895 (PMC9359539; doi:10.1371/journal.pone.0271895)
Supplement: S1 Table — (DOCX) [file pone.0271895.s001.docx]

**Table 1.** Socio demographic clinical data collection sheet

| **Variables** | **Category** | **Frequency** | **Percentage** |
| --- | --- | --- | --- |
| **Sex** | Male |  |  |
|  | Female |  |  |
| **Age** | 18-45 |  |  |
|  | 46-65 |  |  |
|  | >65 |  |  |
| **Residence** | Urban |  |  |
|  | Rural |  |  |
| **Year** | 2015 |  |  |
|  | 2016 |  |  |
|  | 2017 |  |  |
|  | 2018 |  |  |
|  | 2019 |  |  |
|  | 2020 |  |  |
|  | 2021 |  |  |
| **Stage of the Disease** | Stage I |  |  |
|  | Stage II |  |  |
|  | Stage III |  |  |
|  | Stage IV |  |  |
| **Anatomical site of Cancer** | Right Breast |  |  |
|  | Left Breast |  |  |
|  | Both |  |  |
| **Metastasis** | Metastasis to Liver |  |  |
|  | Metastasis to Lung |  |  |
|  | Metastasis to Bone |  |  |
|  | Metastasis to Lymph node |  |  |
| **Co-infection** | Co-infection to IP |  |  |
|  | Co-infection to DM |  |  |
|  | Co-infection to Toxic Goiter |  |  |
|  | Other co-infection |  |  |
